# Development of a Lateral Flow Immunoassay (LFIA) to Screen for the Release of the Endocrine Disruptor Bisphenol A from Polymer Materials and Products

**DOI:** 10.3390/bios11070231

**Published:** 2021-07-11

**Authors:** Anna Raysyan, Rudolf J. Schneider

**Affiliations:** 1 BAM Federal Institute for Materials Research and Testing, 12205 Berlin, Germany; raysyan.anna@gmail.com; 2 Department of Chemistry, Humboldt-Universität zu Berlin, 12489 Berlin, Germany; 3 Faculty III Process Sciences, Technische Universität Berlin, 10623 Berlin, Germany

**Keywords:** LFIA, immunoassay, bisphenol A, endocrine disruptor

## Abstract

One of the most important chemicals used in the production of polymer plastics and coatings is bisphenol A. However, despite the large number of studies on the toxicity and hormonal activity of BPA, there are still open questions and thus considerable media attention regarding BPA toxicity. Hence, it is necessary to develop a sensitive, simple, cost-efficient, specific, portable, and rapid method for monitoring bisphenol A and for high sample throughput and on-site screening analysis. Lateral flow immunoassays have potential as rapid tests for on-site screening. To meet sensitivity criteria, they must be carefully optimized. A latex microparticle-based LFIA for detection of BPA was developed. The sensitivity of the assay was improved by non-contact printing of spot grids as the control and test lines with careful parameter optimization. Results of the test could be visually evaluated within 10 min with a visual cut-off of 10 µg/L (vLOD). Alternatively, photographs were taken, and image analysis performed to set up a calibration, which allowed for a calculated limit of detection (cLOD) of 0.14 µg/L. The method was validated for thermal paper samples against ELISA and LC–MS/MS as reference methods, showing good agreement with both methods.

## 1. Introduction

The rapid increase in the global population has accelerated the production of food, industrial products, and also the need for service activities. This has, in turn, contributed to the massive growth of the corresponding industrial sectors to meet the high demand for goods of all kind. Another effect is that the number of chemicals used in consumer products is steadily increasing, whereas understanding of human exposure to them and associated human health risks often lag behind. Numerous studies on exposure to those chemicals have proven adverse health effects both to humans and animals. Some of the substances used in plastics production show effects on the hormone system and are therefore suspected to contribute to various diseases.

Bisphenol A is one of the most important bulk chemicals in the world, used in the production of polycarbonate plastics and epoxy-based resins. The global volume of BPA use for different application areas is growing and expected to reach 10.6 million tons in 2022 [1]. However, despite the large number of studies on the toxicity and hormonal activity of BPA [2,3,4], there are still open questions and thus considerable media attention regarding BPA toxicity.

Many conventional analytical methods are very sensitive and accurate, yet technically complex, time-consuming, require costly and sophisticated instrumentation, and do not allow for field portability or high-throughput analysis [5,6,7]. The lateral flow immunoassay is a user-friendly format in terms of simplicity and cost-effectiveness and allows for rapid on-site testing [8].

The publications on lateral flow immunoassays for quantitative detection of BPA can be divided into four groups by their detection strategy (optical, thermal, magnetic, electrochemical). Innovative materials have been employed in order to obtain a sensitive and quantitative detection, e.g., special gold nanoparticles [9], a cadmium(II) porphyrin modified carbon paste electrode [10], a black phosphorus/Au material [11], magnetic nanoparticles [12], graphite-like carbon nitride-laden gold nanoparticles [13], and gold nanoparticles with high SERS performance [14]. These amplification strategies have been used as signaling probes to replace the gold nanoparticles and thus improve LFIA sensitivity. However, operating with most of these materials and detection strategies requires access to well-equipped, specialized laboratories and involves instrumental readers to register the signal. Good overviews of advances in BPA detection by LFIA have been given by Nguyen et al. (2016) [15] and Sun et al. (2002) [16].

In our study, we wanted to develop a simple LFIA for screening purposes that is easy to produce and use.

Lateral flow methods based on latex microparticles decorated with antibodies (LMP-IgG) offer several advantages, such as excellent visual evaluation due to intensely blue-colored bands, long-term stability, inexpensiveness, and fair commercial availability [17].

In the present study, we have developed a low-cost, flexible, rapid, and easy-to-use LFIA strip test based on latex microparticles decorated with an BPA-selective antibody for determination of the endocrine disruptor bisphenol A. The analysis can be performed within 10 min, evaluated by the naked eye or by taking a photograph and performing image analysis. In our study, we developed a reproducible LFIA assay, which is cost effective in terms of development and implementation and quantification of the results by readily available software.

## 2. Materials and Methods

### 2.1. Chemicals

The LFIA test strips were cut using a paper cutter (Dahle 502, Novus Dahle GmbH, Lingen, Germany). High-binding 96-well, flat-bottom microtiter plates (MaxiSorp™) were from Nunc (Thermo Scientific, Waltham, MA, USA). PD-10 columns were from GE Healthcare (Munich, Germany).

The anti-bisphenol A mouse monoclonal antibody was provided by the lab of Professor Chuanlai Xu [18] (School of Food Science & Technology, State Key Lab of Food Science and Technology, Jiangnan University, Wuxi, Jiangsu, China), dissolved in a PBS-based buffer (7 mg/mL in 0.01 M PBS, pH 7.4, 1% BSA, 1% glycerol, 0.02% azide). The polyclonal goat anti-mouse HRP-labeled antibody (clone A4416) (whole molecule, 0.5–3 mg/mL in 0.01 M PBS, pH 7.4, 1% BSA, 0.02% azide) and sheep anti-mouse IgG, 0.6–2 mg/mL in 0.01 M PBS, pH 7.4, 1% BSA, 0.02% azide) were provided by Merck Millipore (Darmstadt, Germany).

N-Hydroxysuccinimide (NHS), N-hydroxysulfosuccinimide (Sulfo-NHS), 1-ethyl-3-(3-dimethylaminopropyl)carbodiimide * HCl (EDC), 2-(N-morpholino)ethanesulfonic acid (MES), bovine serum albumin (BSA), bis-phenol A (BPA), bisphenol A-d16 (BPA-d16), bisphenol valeric acid (BVA), bisphenol B (BPB), bisphenol E (BPE), bisphenol F (BPF), bisphenol S (BPS), 4-cumylphenol (4-CP), 4-octylphenol (OCP), and 4-nonylphenol (4-NP) were purchased from Merck KGaA (Darmstadt, Germany). Carboxyl-modified dyed polystyrene microspheres (–COOH) K 030 with diameter 0.276–0.325 µm (dyed latex) (Estapor^®^, product K1-030 blue) and ethanolamine were provided by Merck Millipore (Darmstadt, Germany). Sample and adsorption pads (AP1002500) were provided by Merck Millipore (Darmstadt, Germany). The nitrocellulose (NC) membrane, type CNPF, 10 µm, the conjugate pad PT-R6, and the adhesion poly -vinyl chloride backing pad were obtained from Advanced Microdevices PVT Ltd. (MDI, Ambala Cantt, India).

Absorbance in microtiter plates was measured photometrically with a SpectraMax^®^ Plus384 spectrophotometer from Molecular Devices (Ismaning, Germany) controlled by SoftMax^®^ Pro software (v 5.2). Microtiter plate washing was performed on an automatic ELx405 Select™ microplate washer (BioTek Instruments, Bad Friedrichshall, Germany). Incubation steps for microtiter plates were performed by shaking on a Titramax 101 plate shaker (Heidolph, Schwabach, Germany). All proteins were deposited onto the membrane strips using a sciFLEXARRAYER S3 piezoelectric spotter (Scienion AG, Berlin, Germany). Photographic images were taken with a Canon EOS 750D camera (Canon, Tokyo, Japan) under an LED ring lamp (AIXPI, Shenzhen, China). Quantitative picture analysis of the lateral flow immunoassay photographs was performed with the software Gwyddion (v2.19, Czech Metrology Institute, http://gwyddion.net, accessed on 8 July 2021).

### 2.2. Preparation of the Hapten–Protein Conjugate

Since BPA does not have reactive functional groups, such as carboxylic or amino groups, a commercially available reagent, 4,4-bis(4-hydroxyphenyl)valeric acid (BVA), which is a structural analogue of BPA, was used as a mimotope. They have the same antigenic determinant which, coupled to a protein, can mimic the epitope to which certain anti-BPA antibodies will bind.

The hapten–protein conjugate was produced according to Schmidt et al. [19] with a six-carbon aliphatic chain, which is considered to be the optimal spacer length for enabling optimal binding. The synthesis of functionalized derivatives of the target compound is key to increase the sensitivity of the assay [20]. For this purpose, aminohexanoic acid (Ahx) was coupled with 4,4-bis(4-hydroxyphenyl) valeric acid (BVA). Briefly, BVA (26.3 mg) and NHS (12.9 mg) were mixed with EDC (22.3 mg) and dissolved in 3 mL DMSO in an amber glass vial, and the mixture was incubated with continuous stirring for 2 h at room temperature under argon. Ahx (9.8 mg) was dissolved in 1.5 mL DMSO and was mixed with 1.5 mL PBS (pH 6). Then, 2.5 mL of the reacted solution of BVA was added dropwise to the Ahx solution and incubated for 4 h at room temperature. Because BVA-Ahx is not stable, the carboxylic acid terminus was in situ activated by NHS/EDC chemistry as described above.

A total of 22 mg of BSA was dissolved in 2 mL PBS pH 6. The activated BVA-Ahx was added dropwise to the protein solution. After 4 h reaction time, the protein conjugate was purified via size-exclusion chromatography (SEC) on a PD-10 column, reaching a final protein concentration of BVA-Ahx-BSA (Figure S1A) of 2.5 mg/mL, determined by Bradford assay [21]. The efficiency of the conjugation reaction was verified by MALDI-ToF-MS (Figure S1B).

### 2.3. Antibody Immobilization on Latex Microparticles

The preparation of the LMP-IgG conjugate was done using the protocol from our previous work [22]. Briefly, to 1 mL of 50 mM MES coupling buffer of pH 6.0, 100 µL of carboxy latex beads were added under stirring; subsequently, 24 µL of 200 mM EDC and 240 µL of 200 mM Sulfo-NHS in 1 mL activation buffer (50 mM MES, pH 6.0) were added [23]. After 30 min, the LMP were centrifuged at 14,000 rpm and 10 °C for 7 min, and then the pellet was dispersed in 1 mL activation buffer. To fully disperse the particles, the mixture was sonicated for 2 min. A varied amount of primary antibody stock solution was added to the LMP dispersion and mixed gently. The dispersion was incubated 2.5 h at RT. Then the quenching solution of 30 µL ethanolamine was added and incubated for 30 min. The mixture was centrifuged at 14,000 rpm and 10 °C for 7 min. After centrifugation the microparticles were resuspended in blocking buffer (50 mM Tris with 0.5% BSA, pH 8.0).

### 2.4. Assembly of Lateral Flow Test Strips

**Figure 1:**
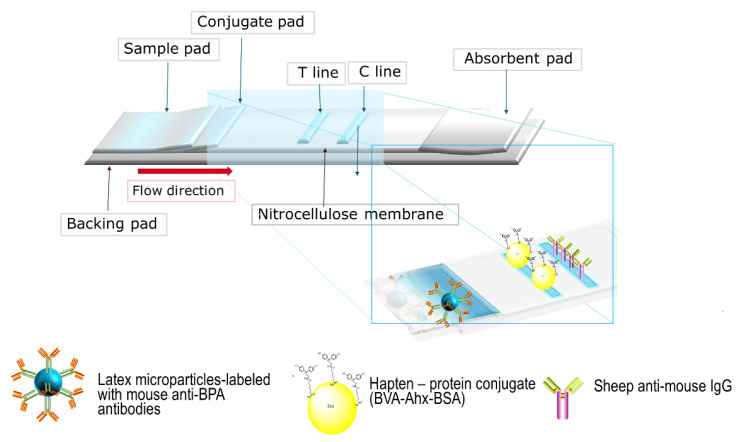
Schematic image of detection of bisphenol A in lateral flow immunoassay.

An LFIA strip consists of a nitrocellulose membrane (NC) with several pads: backing pad, absorbent pad, conjugate pad, and sample pad. The configuration is shown in Figure 1. Onto the NC membrane was deposited the hapten–protein conjugate BVA-Ahx-BSA in the test (T) line zone, and sheep anti-mouse IgG was spotted into the control (C) line zone. An XYZ piezoelectric non-contact spotter was used for this purpose [24]. The non-contact spotting protocol was published by us previously [22]. Briefly, to create the lines with optimum width of the testing zones of around 0.5–1.0 mm, several rows of spots spaced at a distance of 250 µm from each other were applied. Each spot was formed from 40 droplets (each of 0.35 nL) (Tables S1 and S2). The space between test and control lines was 4 mm. The spotted membranes were dried for 1 h at 37 °C. To prevent any nonspecific binding, the membrane was blocked with 1% casein in PBS for 10 min and washed twice with Milli-Q water and dried again for the next hour. Finally, the conjugate pad was impregnated with 5 µL of latex microparticle antibody conjugate (IgG−LMP). The conjugate pad had previously been treated with 20 mM borate buffer (BB) of pH 8.2, containing 1% sucrose and 1% casein. The sample and absorbent pads were laminated to the bottom and upper section of the adhesive backing pad with 2 mm overlap of the NC membrane (Figure 1).

### 2.5. Lateral Flow Immunoassay (LFIA)

The principle of the lateral flow method is based on the competitive binding of bisphenol A from the sample or calibrator and the hapten–protein conjugate (BVA-Ahx-BSA) on the test line (T) with the limited amount of anti-BPA antibodies on the particles (LMP-IgG). If bisphenol A is absent in the sample, the hapten conjugate will capture the antibody together with its particle payload, and this generates a blue line, which can be qualitatively judged by the naked eye. Contrarily, if bisphenol A is present in the sample, it will compete with the hapten conjugate BVA-Ahx-BSA for the limited number of antibody binding sites, leading to a decrease in intensity of the blue line. Invariably, the secondary antibodies in the control line zone will bind the LMP-IgG, creating a line, a control for the validity of an individual run.

### 2.6. Lateral Flow Immunoassay Data Processing

**Figure 2:**
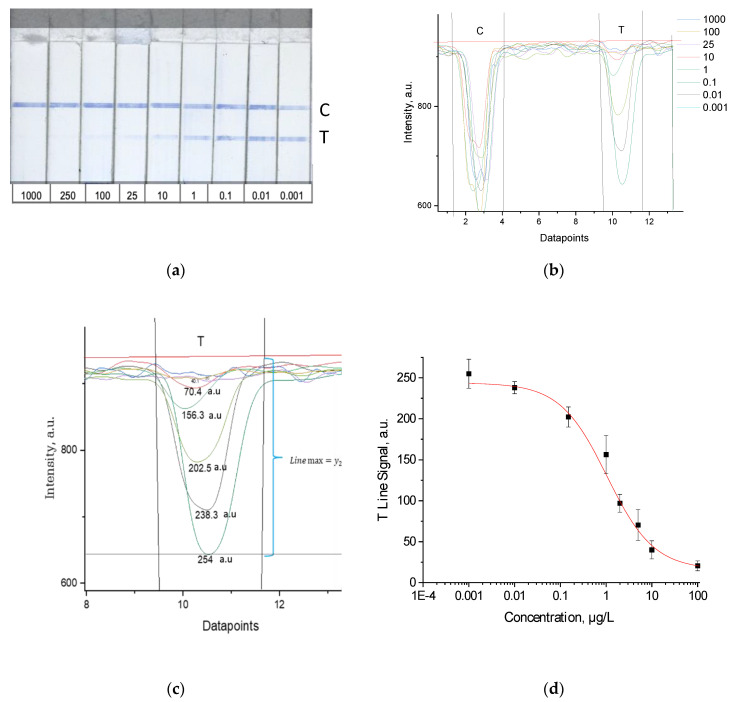
(**a**) Test strips with different concentration of BPA (in µg/L); (**b**) intensity profile of a test and control line and different concentrations of BPA; (**c**) evaluation of different test (T) line signals and baseline correction. The red horizontal line is the baseline for all signals (y_2_). The black horizontal line (at y_1_) is the example for concentration 0.001 µg/L (the maximal intensity (darkest) line, “Line max”, with the deepest intensity dip), the T Line Signal calculated as indicated by subtraction of the intensity values y_2_–y_1_ = 254 a.u. (**d**) Calibration curve obtained by plotting the mean of 3 replicates (n = 3) against BPA calibrator concentrations. The standard deviation (SD) was calculated from the replicates and is shown as error bars.

Calibration solutions of bisphenol A were prepared from a 1 mg/mL stock solution in ethanol by serial dilutions in Milli-Q water. The test strip was inserted into a well of the microtiter plate with 100 µL of BPA calibrator or a real sample extract. After incubation for 10 min at RT, the result was determined both by the naked eye and, after taking a photograph, by the image analysis software Gwyddion (v 2.19) [25]. As shown in Figure 2a, with increasing concentrations of bisphenol A, the color intensity of the test line decreased. The nearest lower concentration of BPA providing still a visible coloration of the test line was taken as the visual limit of detection (vLOD). The color intensity of the lines was evaluated with the help of Gwyddion software (Figure 2b,c). The relation of color intensity and BPA concentration was plotted to raise a calibration curve (Figure 2d). The equation for the calibration curve interpolation (4-parameter sigmoidal fit) was as follows:

$$y=\frac{A-D}{1+{\left(\frac{x}{C}\right)}^{B}}+D$$

where *x* is the analyte concentration and *y* the color intensity. A is the asymptotic maximum of the curve, D is the asymptotic minimum of the curve (background), C is the respective concentration coordinate of the inflection point of the curve (similar to IC_50_, the concentration where 50% reduction of the color intensity is reached), and B is a slope parameter [26,27]. From the signal of a blank sample (without analyte), minus three times the standard deviation of that signal the limit of detection was calculated based on three instrumental readings, which here is called cLOD.

### 2.7. Competitive indirect ELISA

Optimal dilutions of BVA-Ahx-BSA and anti-BPA mouse IgG, respectively, were determined by checkerboard titration. Clear, high-binding microtiter plates were filled with 200 µL per well of the hapten–protein conjugate (BVA-Ahx-BSA) in PBS, pH 7.6. Coating was achieved by shaking with 750 rpm on an MTP shaker overnight at RT. After washing, the plates were blocked using casein in PBS (1%, *w*/*v*, 200 μL per well) for 1 h. The plates were washed again, and 100 µL of sample or BPA standards and diluted anti-BPA monoclonal IgG were added and incubated for 60 min. After a three-cycle washing step, 100 μL peroxidase-labeled goat anti-mouse IgG, diluted 1:20 000 in PBS, were added to each well and incubated for 60 min. After another washing step, 8.5 μL H_2_O_2_ 30% and 550 μL TMB solution (40 mM TMB/8 mM tetrabutylammonium borohydride in N,N′-dimethylacetamide) were added to 22 mL of citrate buffer pH 4.0 (220 mM potassium dihydrogen citrate, 0.5 mM sorbic acid potassium salt) as substrate solution, of which 100 µL were added to each well. After 20 min incubation, the reaction was stopped by adding 100 μL/well of 1 M sulfuric acid. Between plate filling steps, washing steps were performed with a washing buffer (phosphate-buffered saline, PBS, pH 7.6: 0.75 mM potassium dihydrogen phosphate, 6.25 mM dipotassium hydrogen phosphate, 0.025 mM sorbic acid potassium salt, 0.05% (*v*/*v*) Tween™ 20). Absorbance was measured photometrically with the microplate reader at 450 nm and referenced to 620 nm.

### 2.8. Sample Preparation

Thermal paper samples (n = 3) were collected from local supermarkets of different countries: Germany (Berlin), Belgium (Brussels), and Russia (Moscow). Other paper products (n = 9) were collected mainly in Berlin in 2018 (see Table 3). These samples were grouped into 3 categories: flyers (e.g., advertisement brochure from local restaurants), tickets (e.g., train tickets), and food contact papers.

The extraction of bisphenol A was carried out by two methods. In the first method, used for thermal receipt paper, 0.2 g of the thermal paper was inserted into 20 mL of ethanol and stirred for one hour at 35 °C [28]. The second method (taken from [29]) was used for flyers and magazines; 0.1 g of the sample was inserted into 2 mL of methanol and extracted in an ultrasonic bath at 35 °C for ten minutes. The samples were first filtered to remove the resulting dye coagulation and then cleaned with SPE for LC–MS/MS analysis, following a protocol described in literature [30]. For the immunoassay, the solutions were filtrated and diluted in Milli-Q water.

## 3. Results

The performance of different types of nitrocellulose (NC) membrane with different pore sizes (8–15 µm) and different protein binding capacity (low, medium, high), respectively, were evaluated. The CNPF 10 µm membrane was chosen for its good functionality when compared with the other NC membranes. Spotting conditions of the membrane were optimized. The influence of spotting parameters on the final characteristics of the assay has been reported by us previously [22]. In this specific case, optimization improved the limit of detection for BPA by a factor of roughly 100.

### 3.1. Analytical Characteristics of the LFIA

The results of the LFIA test strip can be evaluated by both the naked eye and by photography and image analysis. Visual assessment is convenient for on-site analysis but provides only a qualitative yes/no response around a threshold concentration (vLOD). Moreover, the limit of detection determined by photography and image processing is much lower than the cut-off concentration estimated by naked eye. The instrumental LOD is calculated (-> cLOD) from the standard curve and the mean intensity of the test line for a blank, as described above. Table 1 compares vLOD and cLOD.

For quantification of the color intensity of test and control lines, the photographs of the LFIA strips were processed with the image analysis software Gwyddion (v2.19) (Figure 2). It works with 48-bit images, with intensity values (shades of grey) from 0 to 65,535, and automatically calculates the value in the area in question, which ensures an automated quantitative analysis which is also user-friendly. A gray scale optical analysis was performed from photographed images by the Canon camera under illumination by the LED ring lamp, from which an area of the size 8 × 1.2 mm was selected for the analysis of each image. The program has a function of automatically smoothing the data. The data can be directly transferred to Origin^®^ graphing and analysis software (Figure 2). In contrast to ImageJ free image processing software (ImageJ, National Institute of Health; Bethesda, MD, USA), Gwyddion software allows for the quick processing of large amounts of test strips because of the implemented algorithms for data analysis. ImageJ does not provide these functions, and data processing can only be done manually by using, for example, the software Origin.

### 3.2. Selectivity of the Antibody

Immunoassay selectivity was tested by assaying several compounds structurally related to bisphenol A (Table 2). The specificity of the monoclonal antibody and selectivity of the assay was evaluated [31] by determining their IC_50_ values (midpoints, parameter C in the 4-parametric sigmoidal fitting curve~half maximum inhibitory concentration) and calculating their cross-reactivity (CR, in %) as follows:

$$CR_{\%}=\frac{IC_{50}\left(BPA\right)}{IC_{50}\left(\mathrm{test}\;\mathrm{compound}\right)}\times 100\%$$

## 4. Discussion

Under optimized conditions, this latex microparticle-based LFIA allowed BPA to be visually detected at 10 µg/L (vLOD). Image analysis-based quantification resulted in an LOD (cLOD) of 0.14 µg/L.

Cross-reactivity studies, involving structurally related compounds like BVA, 4-CP, BPE, BPF, and BPS, revealed a high selectivity of the used antibody to BPA. Abstraction of methyl groups and hydroxyl groups of the bisphenol A structure results in a pronounced drop in cross-reactivity, while the extension of the methyl group by an additional carbon in the case of bisphenol B (BPB) resulted in a cross-reactivity of about 200%. High cross-reactivity of bisphenol B can be explained by the higher similarity of the compound to the antigen used for immunization. That antibody was most probably produced from BVA directly coupled to a carrier protein to obtain an immunogen. When structurally simpler phenolic compounds, such as 4-octylphenol (OCP) and 4-nonylphenol (4-NP) were evaluated, the color of the test line in the test strip appeared to be of the same intensity as that of the negative control sample. This was assigned a cross-reactivity of <0.1%. 

Lateral flow immunoassays, such as our assay, rely on the recognition/binding of the analyte by antibodies produced via immunization of mammals. Recent work described the interesting approach of detecting bisphenol A through analyzing, by RT-PCR, the expression by a recombinant *E. coli*, modified with a recA promoter [32], and another work reported the production of a transgenic *A. thaliana* strain that carries the estrogen receptor to which BPA can bind, and an anthocyanin reporter system for indicating this binding [33]. These biosensor developments are very promising, yet mostly lack sensitivity, compared to antibody-based systems. A very comparable signal-enhanced LFIA was proposed by Peng et al. [34], with a comparable vLOD of 10 µg/L; yet the cLOD, after picture analysis by ImageJ software, reached only 2 µg/L. An LFIA described by Dzantiev et al. [35], employing latex particles, reached a comparable vLOD (5 µg/L). By read-out with a flat-bed scanner, an astonishingly more than 10.000-fold lower instrumental LOD of 0.3 ng/L was obtained.

An application, to test for BPA release from coated papers, was set up. It involved as samples thermal receipt paper, public transportation tickets, flyers, and food contact papers (Table 3). Validation was done against ELISA and LC–MS/MS as reference methods, with good agreement of the methods. The results of LC–MS/MS and ELISA reflect the progressing ban of BPA use in thermal receipt papers and food contact papers: the latter was free of BPA. The receipts of two countries were “clean” while a receipt from a third country showed around 0.7 µg/L in the extract. The LFIA was not able to detect this low concentration. The extracts of tickets and flyers all contained between 14 and 52 µg/L BPA.

An additional result, not reflected in Table 3, was, that LC–MS/MS analysis revealed that the extracts of some thermal paper samples contained high concentrations of BPS (ca. 270–780 µg/L) (Figure S4), which sometimes substitutes BPA in its applications.

## 5. Conclusions

To our knowledge, this is the first work reporting the development of a latex-microparticle-based LFIA technique for the detection of BPA. Moreover, a new hapten–protein conjugate, including a C6 spacer (Ahx), was employed in conjunction with a commercially available anti-BPA antibody.

The latex microparticle-based LFIA allowed BPA to be visually detected at 10 µg/L (vLOD). In contrast, photo image analysis-based quantification resulted in an LOD (cLOD) of 0.14 µg/L. This limit of detection is far below the current Specific Migration Limit set for bisphenol A by the European Commission (600 µg/L).

Validation results showed a satisfactory performance of the LFIA for a screening application, which also revealed the substitution of BPA by BPS. Considering the toxicity of bis-phenol S and the fact that there are studies on the release from food cans [36,37], but that little is known about transfer rates of BPS from paper to skin, further studies on BPS release from different materials is recommended.

This lateral flow immunoassay is simple and rapid and could be used for simultaneously analyzing a large number of samples. Additionally, the method is inexpensive and highly portable, allowing for on-site analysis, e.g., for the consumer’s own testing for BPA release.

## Figures and Tables

**Table 1:** Parameters of visual and instrumental detection of BPA in the developed LFIA.

Visual Cut-Off, vLOD, µg/L	Instrumental Limit of Detection, cLOD, µg/L
10	0.14

**Table 2:** Cross-reactivity (CR, in %) of the antibody, determined by LFIA.

Abbr.	Chemical Structure	ELISA	LFIA	Abbr.	Chemical Structure	ELISA	LFIA
BPA	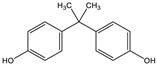	100	100	**BPF**	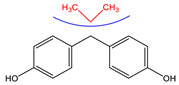	1.0	<0.1
BPA-d16	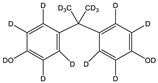	60	52	**BPS**	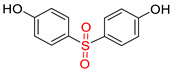	0.13	<0.1
BVA	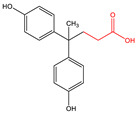	13	17	**BPB**	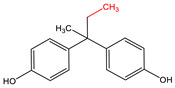	217	105
4-CP	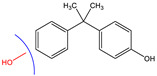	1.6	3.7	**OCP**	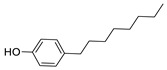	0.01	<0.1
BPE	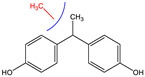	1.0	7.5	**4-NP**	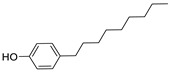	0.02	<0.1

**Table 3:** Analytical results of selected samples (n = 3 replicates). The values of BPA found with LC–MS/MS, ELISA, and LFIA are shown. Information in brackets indicates the recovery rate (BPA in relation to LC–MS/MS) of the immunoassays.

Sample	c (BPA) ± SD				
	LC–MS/MS (µg/L)	LFIA (µg/L)	CV (%)	ELISA (µg/L)	CV (%)
Thermal receipt paper #1	0.70 ± 0.03	<LOD	−	0.76 ± 0.2 (108)	18
Thermal receipt paper #2	<LOD	<LOD	−	<LOD	−
Thermal receipt paper #3	<LOD	<LOD	−	<LOD	−
Tickets #1	14 ± 1.2	15 ± 10 (109)	6	17 ± 2.1 (125)	12
Tickets #2	34 ± 2.3	29 ± 10 (83)	8	43 ± 1.6 (126)	9
Flyers #3	47 ± 3.0	50 ± 7.1 (105)	10	52 ± 4.4 (110)	11
Food contact papers	<LOD	<LOD	−	<LOD	−

## Data Availability

The data presented in this study are available upon request from the corresponding author.

## Supplementary Materials

The following are available online at https://www.mdpi.com/article/10.3390/bios11070231/s1: (1) Characterization of BVA-Ahx-BSA; (2) Optimization of spotting; (3) Verification of the reaction of BVA with Ahx and formation of BVA-Ahx; (4) ELISA performance; (5) Chromatograms to detect BPA and BPS. With Figures S1–S4.
