# Supplementary material for: Development of a Lateral Flow Immunoassay (LFIA) to Screen for the Release of the Endocrine Disruptor Bisphenol A from Polymer Materials and Products
Source: Biosensors (Basel). 2021 Jul 11;11(7):231. doi: 10.3390/bios11070231 (PMC8301804; doi:10.3390/bios11070231)
Supplement: Supplementary file 1 [file biosensors-11-00231-s001.zip › biosensors-1239078-supplementary.pdf]

biosensors

MDPI

Supplementary Information

Development of a Rapid Immunoassay to Screen for the Release of the Endocrine Disruptor Bisphenol A from Polymer Materials and Products

Anna Raysyan, and Rudolf J. Schneider

1) Characterization of BVA-Ahx-BSA

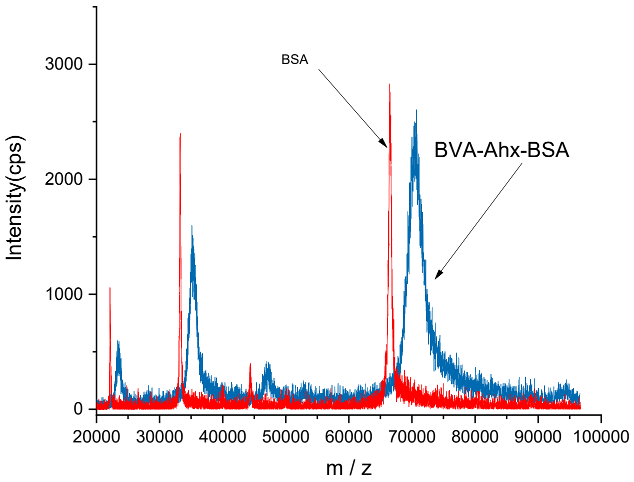

A **B**

Figure S1A Chemical structure of BVA-Ahx-BSA. **Figure S1B** MALDI-TOF/MS spectrum of BVA-Ahx-BSA. Overlay of spectra of unconjugated BSA and BSA conjugated with BVA-Ahx (blue). It can be seen that the mean in the mass signal distribution of the conjugate has increased significantly compared to BSA. The spectra represent the averages of three measurements (n = 3).

2) Optimization of spotting

Our approach to sharper lines was to array nanoliter drops of the reagents, using a non-contact spotter, the spot pitch having to be optimized. Based on earlier experience, solutions were arrayed with 350 µm and 250 µm spot to spot pitch (Table S1). It was found that the distance between the spots with 350 µm spacing is too wide which may have led to inaccurate readings. So, we continued with a spot pitch of 250 µm.

Table S1 Influence of spot pitch on the image of the test line.

Spot pitch 350 µm	Spot pitch 250 µm
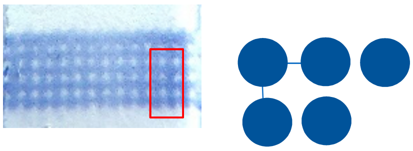	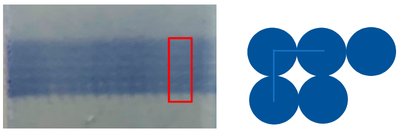

Yet, the signal intensity is also a result of the load of coating antigen (BVA-Ahx-BSA) in the test line zone and the secondary antibody (anti-mouse) in the control line zone and the number of spots. In a specific study, the overall loadings of the BVA-Ahx-BSA hapten protein conjugate as well as the antibody loading were decreased by a factor of 5 from the initial level. As a consequence, the resultant maximal value of the optical signal readout became 2.5-fold lower, from 500 to 250 units. However, due to a reduction from 75 to 30 spots, the mean color intensity of an individual spot remained the same (2.65 units). The optimization allowed for improving the limit of BPA detection 100-fold without loss of visual reading quality (Table S2).

Table S2. Optimization of reagent load and test strip characteristics for BPA detection.

BVA-Ahx-BSA application			Antibody load		Assay characteristics		
Number of droplets / / spotted volume	T-line zone, lines x spots	Load per strip, µg	IgG, µL per ml LMP	IgG–LMP, µL per strip	Max. intensity a.u.	Intensity per spot	Visual LOD, µg/L
40 // 14 nL	5 × 15	0.9	40	5.0	500	6.7	1000
40 // 14 nL	5 × 15	0.9	40	2.5	400	5.4	250
20 // 7 nL	5 × 10	0.4	20	2.5	350	11	25
25 // 8.75nL	3 × 10	0.2	20	2.0	250	8.3	10

3) Verification of the reaction of BVA with Ahx and formation of BVA-Ahx

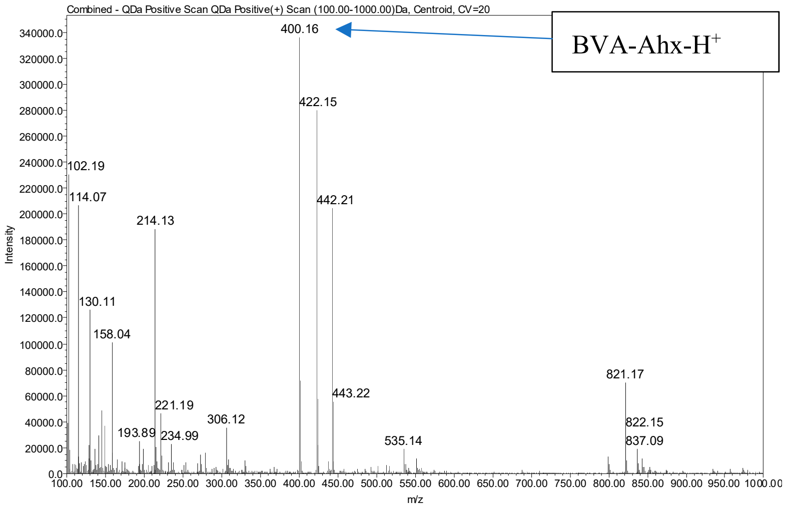

Figure S2 Mass spectrum of BVA-Ahx-H+. Verification of the reaction of BVA with Ahx and formation of BVA-Ahx. The largest signal at m/z = 400.16 can be attributed to BVA-Ahx-H^+ (mass of the desired product of 399 g/mol plus the mass of a proton (+1)).

4) ELISA performance

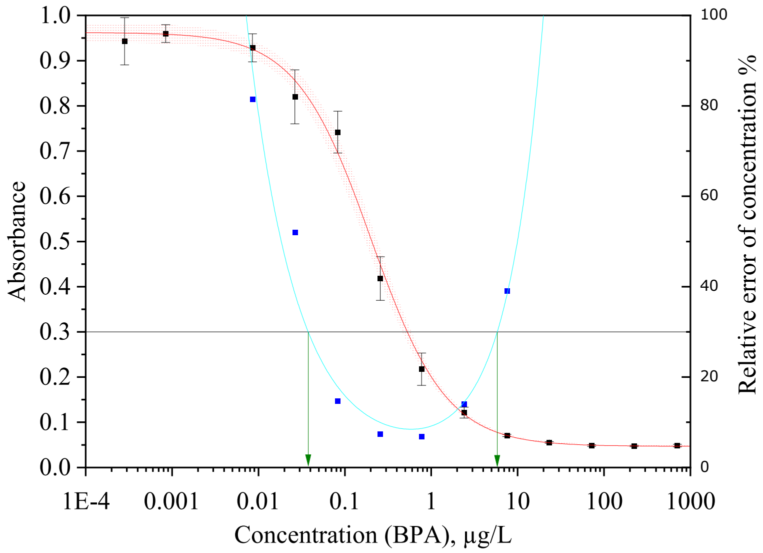

Figure S3 ELISA calibration curve. Indirect assay with coating conjugate BVA-Ahx-BSA. Calibration curve (red solid line), precision profile (blue squares and cyan line), and measurement range (indicated by green arrows) from 0.05 to 8 µg/L (intersection points at 30% maxium allowable relative error of the concentration, solid black lines).

5) Chromatograms to detect BPA and BPS.

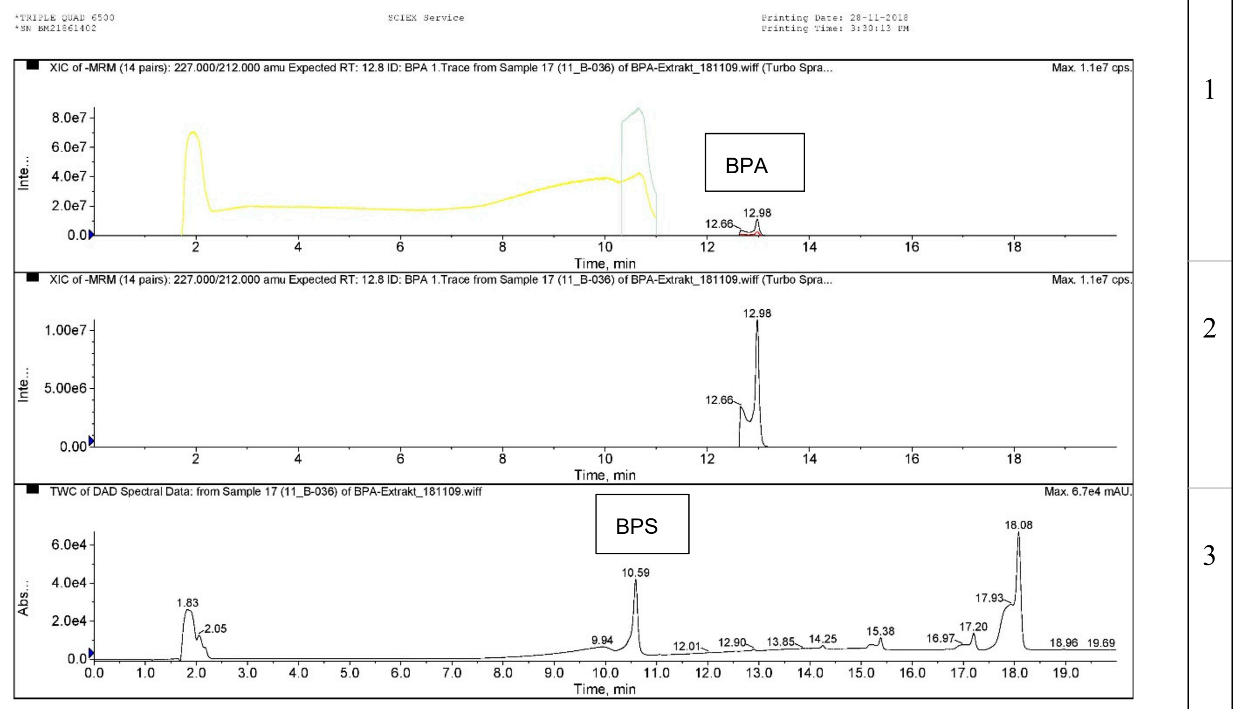

A. Thermal receipt paper BE

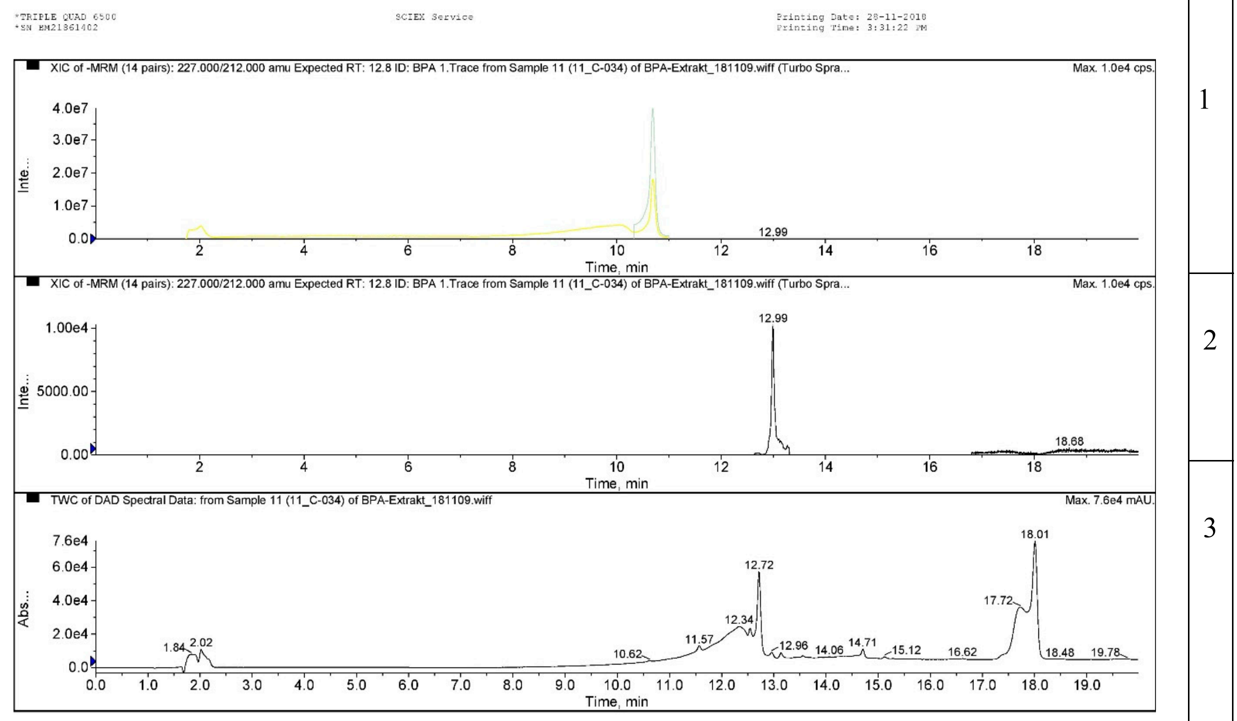

B. Thermal receipt paper DE

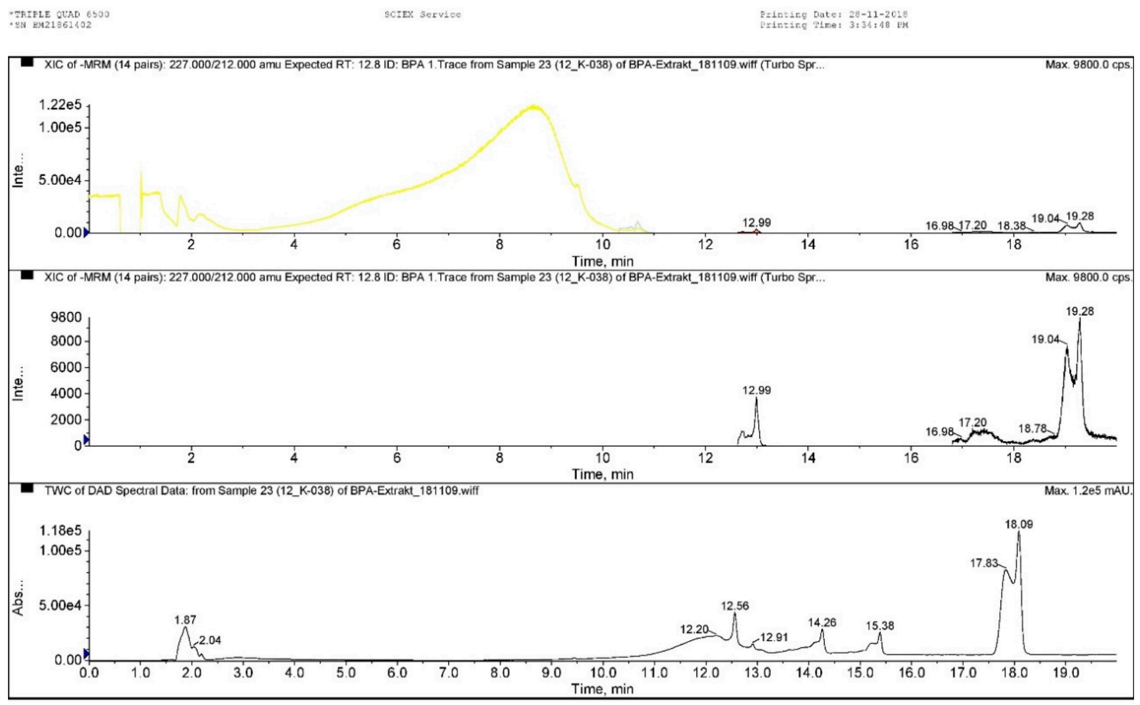

1

2

3

C. Thermal receipt paper RU

Figure S4 Chromatograms to detect BPA and BPS. Extracts from 3 thermal receipt paper samples. 1) XIC (extracted ion chromatogram) for transition m/z = 227->212 with peaks expected for BPS around 10.59 min. and BPA around 12.99 min. 2) XIC, enlarged. 3) DAD trace.
